# Exaggerated Blood Pressure Response to Exercise Is a Risk of Future Hypertension Even in Healthy, Normotensive Young Individuals—Potential Preventive Strategies for This Phenomenon?

**DOI:** 10.3390/jcm13195975

**Published:** 2024-10-08

**Authors:** Narumi Kunimatsu, Hayato Tsukamoto, Shigehiko Ogoh

**Affiliations:** 1 Department of Biomedical Engineering, Toyo University, Saitama 351-8510, Japan; naruntyann@gmail.com; 2 Faculty of Sport Sciences, Waseda University, Saitama 359-1192, Japan; hayato.tsukamoto@aoni.waseda.jp

**Keywords:** exercise pressor reflex, arterial stiffness, cognitive function, cardiovascular disease, exercise hypertension

## Abstract

Physical activity and regular exercise are well known to reduce the risks of cerebrovascular and cardiovascular diseases, leading the American College of Sports Medicine to endorse the concept that “exercise is medicine”. However, a single bout of exercise temporarily raises arterial blood pressure (BP) to meet the metabolic demands of working muscle, and this BP response is particularly exaggerated in older adults and patients with cardiovascular conditions, such as hypertension, resulting in an exaggerated BP response during exercise. This presents a paradox: while regular exercise is crucial for preventing these diseases, excessively high BP responses during exercise could increase the risk of vascular damage. The mechanisms underlying this exaggerated BP response during exercise remain unclear, and effective exercise regimens for these populations have yet to be established. Currently, low-intensity exercise is recommended; however, its efficacy in disease prevention is uncertain. Notably, even among healthy individuals, there is significant variation in the BP response to exercise. Some healthy individuals, despite having normal resting BP, exhibit an exaggerated BP response during physical activity. Importantly, these individuals are often unaware that their BP becomes excessively elevated during physical activity. Repeated exposure to these heightened BP responses through regular physical activity may increase their long-term risk of cardiovascular disease. How can we prevent disease development in these individuals while still ensuring the effectiveness of exercise? Some studies have shown that individuals with a family history of hypertension may experience this phenomenon even in children and adolescents. Additionally, left ventricular hypertrophy contributes to an exaggerated BP response to exercise, suggesting a possible genetic influence. Conversely, other reports indicate that factors such as arterial stiffness, obesity, and low exercise capacity also contribute to this exaggerated response. Our recent preliminary data suggest that the cognitive benefits of exercise may be diminished in individuals who exhibit an exaggerated BP response during exercise. This implies that individuals with an exaggerated BP response, despite having normal resting BP, may not fully benefit from exercise. In this perspective paper, we review the physiological aspects of this phenomenon and explore strategies to address it. Additionally, we discuss BP responses in athletes within this content. Our goal is to prevent disease while maximizing the benefits of exercise for healthy individuals with an exaggerated BP response, as well as for elderly and cardiovascular patients.

Hypertension significantly increases the risks of coronary heart disease, stroke, peripheral artery disease, and other forms of cardiovascular disease (CVD) [1]. Hypertension is the primary modifiable risk factor for preventing CVD and related mortality [2,3], as even a small reduction in blood pressure (BP) can lead to a substantial decrease in CVD events [4,5]. Notably, no significant differences in mortality have been observed between individuals with controlled hypertension and those with normal BP [6]. Thus, effective BP management, especially in patients with hypertension, is essential for preventing CVD. Lifestyle interventions, including enhanced physical activity, improved diet, maintenance of a healthy body mass index, and reduced alcohol consumption, have proven effective in lowering BP [7]. Among these, exercise is particularly emphasized for its role in reducing the risk of CVD, as even small amounts of physical activity have been shown to decrease mortality [8]. A systematic review and meta-analysis of a longitudinal cohort study [9] investigated the relationship between physical activity and incident hypertension, concluding that higher levels of physical activity provide additional protective benefits against hypertension. Moreover, both cardiorespiratory fitness and physical activity levels are inversely associated with the development of hypertension [10]. A significant improvement in cardiorespiratory fitness was linked to a lower risk of incident hypertension compared to individuals who maintained the same fitness levels [11]. A meta-analysis [12] evaluated the relative effectiveness of pharmaceutical versus physical activity interventions, finding that regular exercise was as effective as commonly prescribed medications in the secondary prevention of coronary heart disease. In light of these findings, the American College of Sports Medicine (ACSM) advocates the concept that “exercise is medicine” [13]. This approach supports physicians in encouraging their patients to incorporate exercise into their disease prevention and treatment strategies.

Indeed, chronic aerobic exercise has been shown to produce a significant and clinically meaningful reduction in BP in a dose-dependent manner, with the greatest reduction observed at 150 min/week [14]. However, it is well known that a single bout of exercise increases arterial BP to support muscle metabolism during physical activity. This response is particularly pronounced in older individuals [15] and patients with cardiovascular conditions such as hypertension [16], leading to an exaggerated BP response during exercise.

Consequently, individuals who exhibit an exaggerated BP response to exercise, such as the elderly, and patients with CVD, may face a continuous risk of elevated BP during physical activity. This presents a paradox (Figure 1): while exercise is crucial for preventing cerebral and cardiovascular diseases in these populations, they also face an increased risk of damage to cerebral and cardiac vasculature due to an exaggerated BP response during exercise. As a result, exercise therapy may have limitations for these individuals. The mechanisms underlying this exaggerated BP response to exercise in these populations remain unclear, and appropriate exercise regimens have not been firmly established. Currently, low-intensity exercise or reduced workloads are recommended to mitigate the BP response during exercise for these populations [17]. However, the effectiveness of such low-intensity exercise in disease prevention remains uncertain, given the dose-dependent impact of exercise on BP [14].

Interestingly, there is significant variation in BP response to exercise among healthy individuals [18,19,20,21]. Some healthy individuals with normal resting BP exhibit an exaggerated BP response during exercise [20,22,23]. This exaggerated BP response has been termed “exercise hypertension” when it reaches or exceeds the 90th percentile of age- and sex-specific normative data [24]. Regarding BP response to exercise, the American Heart Association (AHA) suggests that systolic blood pressure (SBP) should increase by ~10 mmHg per metabolic equivalent (MET) during graded exercise [25]. Moreover, the exaggerated BP response during exercise (exercise hypertension) has been defined by several organizations. The AHA considers an SBP of 210 mmHg for men and 190 mmHg for women as concerning during exercise [26,27], while the European Society of Cardiology (ESC) sets higher thresholds of 220 mmHg for men and 200 mmHg for women [28]. The ACSM proposes a unisex threshold of 225 mmHg for both sexes during exercise [29]. While there is no consensus on a specific threshold value of exercise hypertension, a previous study [30] suggests that it could be identified from BP measurements during a single bout of low-intensity exercise. This study proposed that an SBP of ≥175 mmHg during light to moderate exercise (60–70% of age-predicted maximal heart rate for approximately 3–5 min) may indicate an increased risk related to an exaggerated BP response during exercise.

Early detection of ‘exercise hypertension’ is crucial because individuals who are normotensive at rest but exhibit an exaggerated BP response to exercise may be at greater risk of developing hypertension in the future [27,31,32,33,34]. Notably, both isometric and dynamic exercises have been evaluated as predictors of future hypertension [35,36,37]. Several studies [38,39,40,41] have indicated that normotensive participants with an exaggerated BP response to exercise are two to three times more likely to develop hypertension compared to participants with a normal BP response to exercise. Early research by Dlin et al. [42] followed eight subjects who were normotensive at rest (BP ≤ 140/90 mmHg) but showed an exaggerated BP response to exercise (SBP ≥ 200 mmHg and/or diastolic BP ≥ 90 mmHg) over a period of 5.8 years. These subjects developed hypertension, leading the authors to suggest that an exaggerated BP response to exercise may serve as an additional risk marker for hypertension. Additionally, a study by Matthews et al. [31] examined the association between an exaggerated BP response to treadmill exercise and the risk of developing hypertension. Those subjects were healthy normotensive men (*n* = 5386) who had a baseline graded maximal exercise test and completed a mailed follow-up questionnaire. In multiple logistic regression analysis, an exaggerated BP response was found to predict (odds ratio = 3.0, 1.5–6.1) future hypertension, even after adjusting for sitting SBP and diastolic BP. Therefore, an exaggerated BP response to exercise is independently associated with an increased risk of future hypertension.

Early disease detection, risk assessment, and ongoing monitoring are essential for preventing CVD. For instance, evaluating arterial stiffness via pulse wave velocity (PWV) is key to understanding hypertension development. Imaging techniques such as supra-aortic trunk ultrasound, echocardiography, cardiac magnetic resonance, and coronary computed tomography angiography have expanded our understanding of cardiovascular anatomy and function [43]. These methods allow for personalized prevention strategies by providing detailed insights into cardiac and vascular health, significantly enhancing our ability to combat CVD progression. Similarly, exercise hypertension is associated with an increased risk of target organ damage and predicts future cardiovascular events and mortality [27]. Importantly, while PWV and imaging techniques require specialized equipment and trained personnel, evaluating exercise hypertension can be more straightforward, as it only requires measuring BP during exercise. These backgrounds suggest that an exaggerated BP response to exercise could be a useful early indicator of essential hypertension or pre-hypertension [27]. Individuals with exercise hypertension may encounter similar challenges with exercise therapy as do the elderly and patients with hypertension who also exhibit an exaggerated BP response during exercise. However, these individuals often do not realize that their BP becomes excessively high during physical activity because BP measurements are typically not taken during exercise. The purpose of repeated physical activities and exercise is to reduce resting arterial BP and lower the risk of cardiovascular and cerebrovascular diseases. For this purpose, thus, resting BP is essentially measured but not during exercise. More importantly, chronic exercise can repeatedly expose these individuals to exaggerated BP responses, potentially increasing their risk of CVD.

How can we prevent disease development in these individuals who exhibit an exaggerated BP response to exercise despite having normal resting BP, while still ensuring the effectiveness of exercise? Currently, we lack a definitive answer because the mechanism behind this exaggerated BP response remains unclear. To begin addressing this issue, it is crucial to identify the mechanisms responsible for the exaggerated BP response during exercise and to examine the associated physiological factors. Some studies have shown that individuals with a family history of hypertension [44] or those who experienced an exaggerated BP response during childhood and adolescence [22] continue to exhibit this response in adulthood. Additionally, higher left ventricular mass in normotensive individuals has been linked to an exaggerated BP response during exercise [45,46]. Increased left ventricular mass during childhood may also be a significant predictor of future hypertension and its consequences [47]. These findings suggest that genetic factors may play a role in the exaggerated BP response during exercise in individuals with normal resting BP. Conversely, environmental factors such as arterial stiffness [48], metabolic syndrome [49], and low exercise capacity [50] have also been identified as contributors to this exaggerated BP response. Wuttichaipradit et al. [48] have highlighted the impact of arterial stiffness, assessed by the Cardio-Ankle Vascular Index, on excessive exercise-induced SBP response, independent of other established CVD risk factors. Given these complexities, exercise hypertension may result from a combination of genetic and environmental factors, and the precise mechanisms underlying an exaggerated BP response with normal resting BP may not be straightforward. For individuals with an exaggerated BP response during exercise, it is crucial to continue exercising to prevent future hypertension while carefully managing BP not only at rest but also during physical activity. Further research is needed to elucidate the involved mechanisms and to develop targeted exercise interventions that balance the benefits of physical activity with effective BP management.

Exercise hypertension could serve as an early warning signal of abnormal BP regulation that might not be detected through standard clinical BP measurements. However, controlling BP during exercise can be challenging for these individuals. Schults and Shaman [27] have outlined several interventions for managing exercise hypertension. Pharmacological interventions are one such approach. They highlight that various combination therapies and monotherapy antihypertensive agents (including β-blockers, α-receptor blockers, calcium antagonists, angiotensin-converting enzyme inhibitors, serotonin antagonists, and dual-action compounds) can effectively reduce both resting and exercise SBP and diastolic BP, regardless of the drug class. However, individuals with exercise hypertension but normal resting BP may be clinically healthy and thus may not recognize their elevated BP during exercise, limiting their access to these pharmacological treatments. Lifestyle modifications, including regular exercise, are widely recognized as crucial for the prevention and management of hypertension [51]. The beneficial effects of exercise training and lifestyle modification on exercise BP are often more apparent in populations at a higher risk of elevated BP, such as those with essential hypertension, obesity, a sedentary lifestyle, or a history of cerebrovascular disease events [52]. A previous study [53] examined the impact of a 1-year lifestyle intervention (exercise and dietary modification) on exercise BP in patients with type 2 diabetes and found that while exercise training was effective, the lifestyle intervention alone was insufficient to normalize BP responses to exercise. This suggests that more intensive longer-duration exercise interventions may be necessary to achieve BP response during exercise.

Given these circumstances, individuals with an exaggerated BP response still need to engage in exercise to reduce their risk of CVD. However, these individuals may not recognize their elevated risk of CVD since their resting BP is normal. Consequently, it is essential to monitor BP during exercise in addition to resting BP measurements to identify exercise-induced hypertension and mitigate the risk of CVD. Furthermore, exercising at an intensity tailored to an individual’s arterial BP response may be crucial for optimizing the benefits of exercise. The cardiopulmonary exercise test has significant applications in athletes, providing the physiological parameters that determine exercise performance [54]. Because of the relationship between BP variations and oxygen consumption, a cardiopulmonary exercise test should be useful to identify a normal or exaggerated BP response to exercise (exercise hypertension). However, the underlying factors contributing to an exaggerated BP response during exercise in different populations, such as the elderly and patients with CVD, may differ significantly from those in young, healthy individuals. Thus, simple muscle exercises, such as handgrip exercises, and monitoring arterial BP responses during these exercises could serve as useful screening tools for customizing exercise prescriptions to identify and manage exercise-induced hypertension.

Interestingly, our recent preliminary data suggest that individuals with an exaggerated BP response to exercise may not fully experience the cognitive benefits typically associated with exercise. In Figure 2 (our unpublished data), it is demonstrated that exercise improved cognitive function in individuals with low and moderate BP responses. However, those with a higher BP response to handgrip exercise experienced an attenuation of the exercise-induced cognitive benefits. This implies that even with normal resting BP, individuals who exhibit an exaggerated BP response during exercise may not reap the full benefits of physical activity. Furthermore, these findings indicate that when developing exercise protocols to maximize benefits while avoiding hypertensive risks, it is crucial to account for individual physiological variations in arterial BP response. These variations can be influenced by individual factors, as well as exercise intensity, mode, and duration. Specifically, in exercise rehabilitation for individuals with a high arterial BP response, older adults, and patients with hypertension, it is essential to consider the potential impact of an excessive BP response on cognitive function improvement when selecting exercise workload, mode, or duration. Future research should focus on identifying threshold values for increased risk associated with exaggerated BP responses during exercise and on further investigating the underlying physiological factors involved in this phenomenon.

Athletes undergoing intense training often exhibit exaggerated BP responses during exercise, which differ from those of non-athletes. While these responses are apparent, their clinical implications are not yet fully understood [55]. Increased SBP in endurance athletes is typically linked to higher peak oxygen consumption (VO_2max_), greater work capacity, and occasionally increased myocardial mass [56]. Notably, exaggerated BP responses may predict early cardiovascular events or sudden cardiac death, with studies indicating a 3.6-fold increase in the risk of incident hypertension over 6.5 years in athletes [57]. Chronic intensive exercise training induces fibrosis in both atria and increases susceptibility to atrial fibrillation (AF) [58], facilitated by atrial remodeling, atrial ectopy, and an imbalance in the autonomic nervous system [59]. Additionally, a recent meta-analysis [60] revealed a five-fold increase in the risk of AF in endurance athletes. These findings suggest a higher prevalence and relative risk of AF in the athletic population. Previous studies [61] have shown that male non-elite athletes exhibit higher BP at rest and during peak exercise, a more concentric type of left ventricular remodeling, and altered diastolic function, which may contribute to more pronounced atrial remodeling [62]. Thus, the cardiac adaptations in athletes associated with exaggerated BP responses during exercise may increase the risk of CVD. However, in contrast, elevated BP responses in athletes do not consistently correlate with adverse health outcomes [63]. This inconsistency may arise from different cardiac adaptations across sports categories (endurance, power, mixed). However, the physiological relationship between BP response and CVD risk remains unclear in both athletes and young, healthy individuals.

Altogether, an abnormally high BP recorded during exercise testing may serve as a clinically valuable warning signal for physicians, indicating a potential BP abnormality that warrants further investigation. It is essential to further elucidate the underlying physiological determinants of elevated exercise BP. Additionally, gaining a deeper understanding of the role of central hemodynamics in the development of exercise-induced hypertension will be an important area for ongoing research.

## Figures and Tables

**Figure 1:**
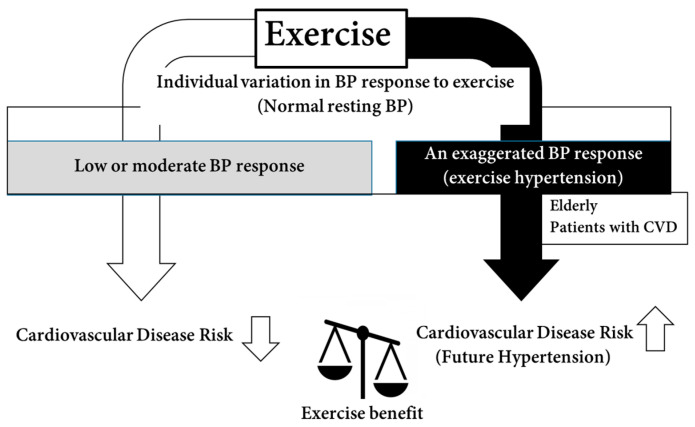
Individuals with an exaggerated BP response to exercise may face sustained BP elevation during activity. This presents a paradox: while exercise helps prevent cerebral and cardiovascular diseases, it also increases the risk of vascular damage due to heightened BP during exercise. BP, blood pressure; CVD, cardiovascular disease.

**Figure 2:**
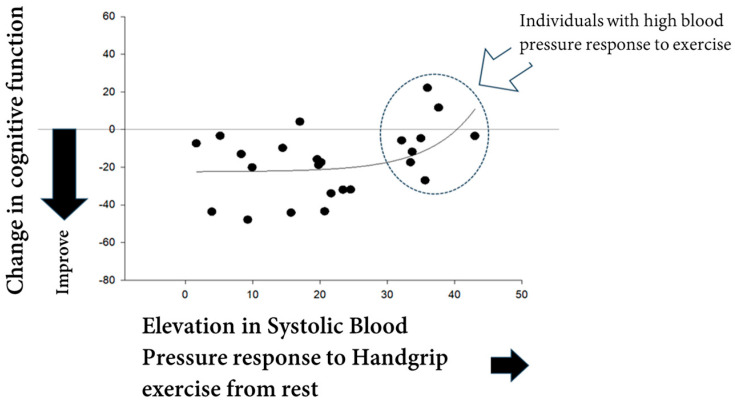
Individual variability in blood pressure response to exercise is evident. While exercise typically improves cognitive function (indicated by negative values), individuals with a higher blood pressure response to handgrip exercise may not experience this cognitive benefit (improvement in cognitive function) (unpublished data). Black dots represent individual data points (changes in systolic blood pressure and cognitive function, reaction time to Go/No-go task), and gray line represents the regression line of all data points following this sentence.

## Data Availability

Not applicable.
